# Upconversion luminescence enhancement by Fe^3+^ doping in CeO_2_:Yb/Er nanomaterials and their application in dye-sensitized solar cells

**DOI:** 10.1039/d0ra02308f

**Published:** 2020-05-19

**Authors:** Jingyi Bai, Pingping Duan, Xin Wang, Gui Han, Min Wang, Guowang Diao

**Affiliations:** School of Chemistry and Chemical Engineering, Yangzhou University Yangzhou 225002 PR China jybai@yzu.edu.cn gwdiao@yzu.edu.cn; Guangling College, Yangzhou University Yangzhou 225002 PR China

## Abstract

To make use of broad spectrum solar energy remains a main target in the photoelectrochemical area. Novel promising photoelectrode CeO_2_:Fe/Yb/Er nanomaterials supported on upconversion nanomaterials doped with transition-metal ions are reported to improve broad spectrum absorption and scattering properties in dye-sensitized solar cells (DSSCs) for the first time. The results demonstrate that the materials have stronger upconversion luminescence than CeO_2_:Yb/Er samples when the Fe^3+^ ion doping concentration is 2 mol% and 33.5% higher photoelectric conversion efficiency than a pure P25 electrode, which are attributed to the special light scattering properties and excellent dye adsorption capacity of the CeO_2_:Fe/Yb/Er nanomaterials. Accordingly, doping Fe^3+^ transition metal ions in the upconversion material CeO_2_:Yb/Er provides a new research idea for improving the photoelectric conversion efficiency of DSSCs.

## Abstract 2

The Fe^3+^-doping in CeO_2_:Fe/Yb/Er enhances upconversion luminescence by destroying the symmetry of the crystal field around Er^3+^, which improves DSSC performance.

## Graphical abstract

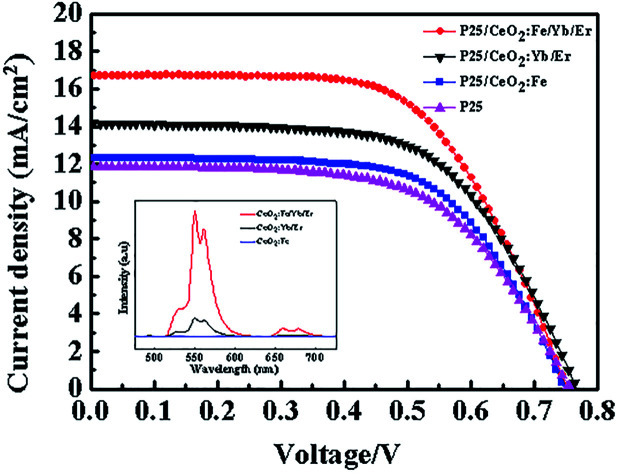

## 1. Introduction

In today's deepening energy crisis, the inexhaustible solar energy has received more and more attention because of its safety, sustainability, and carbon-free nature.^1^ In the photovoltaic industries using solar energy, dye-sensitized solar cells are photoelectrochemical systems with dye-adsorbed porous-structured oxides as photoanodes, which have the advantages of low cost, high efficiency and good stability.^2^ However, the problem of spectral mismatch between the incident solar spectrum and the band gap of the semiconductor photoanode greatly reduces the utilization of sunlight by the battery. In particular, the near-infrared (NIR) light which accounts for nearly 50% of sunlight is wasted. Hence, enlarging the range of absorbable wavelengths in sunlight is one of the effective measures to reduce energy loss.^3^

In order to obtain higher photocurrent values in the NIR region, researchers turned their attention to up-conversion materials that can convert low-energy excitations into high-energy emissions. Up-conversion nanophosphors (UCNPs) with nonlinear optical effects can convert NIR light into visible light by means of energy transfer or multiple absorptions.^4^ Then the dye molecule absorbs visible light and produces more electrons, which promotes the conversion of sub-band gap photons into above-band gap photons to decrease transmission loss. Consequently, it is feasible to improve battery performance by introducing UCNPs into solar cells to enhance their utilization of sunlight.^5^

In order to realize the above potential applications, it is particularly important to find a suitable matrix material. The semiconductor oxide material CeO_2_ has been widely in many fields such as photocatalysis, water oxidation, phosphors and thermoelectric materials, which is due to its environmental friendliness, thermal stability and chemical stability.^6,7^ Especially in this work, the similar ionic radius of Ce^4+^ and Yb^3+^, Er^3+^ are conducive to the doping of rare earth ions and the lower phonon energy of CeO_2_ is favorable for the emission of UC by reducing the multiphonon relaxation, which all lay the foundation for CeO_2_ as the host material.^8,9^ On the other hand, the remarkable features of CeO_2_ such as high refractive index and high optical transparency provide support for its use as a light scattering layer to increase light collection efficiency in DSSCs with double-layer photoanode.^10^ Herein, we have chosen CeO_2_ as the host material for UCNPs is reasonable, and it is also the unique innovation of our team based on the above two considerations.^11^ But the current exploration of CeO_2_-based up-conversion materials is mostly achieved by the doping between Er^3+^/Yb^3+^ and Er^3+^ rare earth ions, which makes it difficult to further improve the luminous intensity. Thereupon, how to overcome this problem becomes a top priority.

As we all know, the probability of electronic transition has a decisive influence on the intensity of upconversion luminescence in the UCNPs, but it is also easily affected by the local crystal field around the RE ions.^12^ In the sense, in addition to utilizing the plasma effect, photonic crystal effect and constructing a core–shell structure, it is a great significance to increase the UC efficiency of UCNPs by changing the symmetry of local crystal fields.^13^ In published literature reports, researchers have used this approach to achieve their goals. For example, the green light emission enhanced by 34 times and red light emission enhanced by 101 times when the Li^+^-doping concentration was 0.5 mol%, which were found in Wang's studies.^14^ The three-doping system with Mg^2+^ ions can significantly increase the red, green and purple UC emissions in UCNPs by Zhao's research.^15^ Compared with alkali ion doping, transition metal (TM) ions have potential to adjust the excited state properties to match the acceptor ions because the exposure of d electrons renders them very sensitive to the environment. Especially, the TM ions Fe^3+^ have many energy levels to enable more energy exchange with RE ions.^16^

The use of Fe^3+^ ions alone in batteries has a number of drawbacks, so we have introduced Fe^3+^ into DSSCs by co-doping. This paper is the first time to explore the effect of Fe^3+^ on the strength of UC in CeO_2_ matrix materials, which has important reference significance. The incorporation of non-rare earth ion Fe^3+^ will adjust the symmetry of the local crystal field in the materials to enhance UC luminescence intensity. The prepared materials were used as photoanodes for dye-sensitized solar cells, and the good photovoltaic performance was obtained. At the same time, a new approach has been opened for the application of Fe^3+^ ions in DSSCs.

## 2. Experiments

### 2.1 Preparation of CeO_2_:Fe/Yb/Er

0.8 g polyvinylpyrrolidone (PVP, K-30) was ultrasonically dispersed into 30 mL ultrapure water, and magnetically stirred with 60 minutes to form a clear solution. Subsequently, 2.4 mmol of metal ions [(96.7 − *x*) mol% Ce^3+^, 0.3 mol% Yb^3+^, 3 mol% Er^3+^, *x* mol% Fe^3+^ (*x* = 0.5, 1, 2, 3, 5)] was mixed with PVP solution and stirred vigorously for 60 minutes to make the mixture uniform. The homogenized liquid was poured into a Teflon-lined with 50 mL and hydrothermally treated at 200 °C for 12 hours. The product was centrifuged at 8000 rpm min^−1^ and washed several times with deionized (DI) water and ethanol, then dried overnight at 80 °C. Moreover, the precipitate was placed in a muffle furnace, heated at 400 °C for 1 hour, and then the same heating rate was controlled to continue to rise to 1100 °C and held for 3 hours. In addition, the SiO_2_ layer was wrapped on the surface of the materials to protect its morphology at high temperature; it was eliminated with NaOH solution (1 M) after calcination. For comparison, the CeO_2_:Yb/Er and CeO_2_:Fe nanomaterials were prepared by the same method.

### 2.2 Fabrication of solar cells

**Fig. 1:**
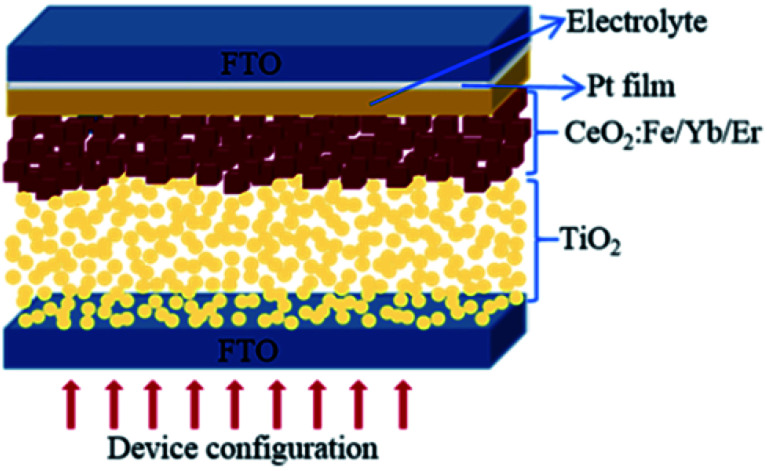
The schematic configuration of DSSCs.

For the double layer photoanode, P25 paste was screen-printed on the FTO glasses, then the CeO_2_:Fe/Yb/Er nanoparticles were coated as an upper film on the basis of the TiO_2_ film. The double layer films with area of 0.16 cm^2^ were sintered at 450 °C for 30 minutes to form the photoanodes, which will be immersed in N719 dye solution (4.5 × 10^−4^ M in absolute acetonitrile) for 24 hours. Subsequently, rinsed photoanodes with absolute ethanol and allowed to air dry. The Pt-coated counter electrode and working electrode were assembled to form a sandwich-type battery whose structure is shown in Fig. 1. Finally, 0.5 M *tert*-butylpyridine, 0.1 M LiI, 0.60 M BMII and 0.05 M I_2_ were dissolved in an acetonitrile solution to obtain the I^−^/I_3_^−^ electrolyte, which was dropped into the battery.^17^ For comparison, pure P25 electrode, CeO_2_:Yb/Er electrode and CeO_2_:Fe electrode were also assembled.

### 2.3 Characterization

The X-ray diffraction which used the Cu-Kα radiation (*λ* = 1.5406) has characterized the phase purity of powder. And the overall structure was studied by scanning electron microscopy (FE-SEM) and transmission electron microscopy (TEM). High-resolution transmission electron microscopy (HR-TEM), Energy dispersive X-ray (EDX) and X-ray photoelectron spectra (XPS) have analyzed the atomic composition of the sample. The Fluorescence spectrophotometer (Shimadzu 200) measured all spectra of upconversion photoluminescence emission by using a 980 nm semiconductor laser as the excitation source, because it has steady state fluorescence. The diffuse reflectance spectra of sample were observed by a UV-vis-NIR spectrophotometer (Cary 5000). Dye desorption measurements were carried out by detaching the N719 dye from photoanode films in NaOH solution (0.1 M) and the concentration has been evaluated by UV-vis absorption spectroscopy. The *I*–*V* and the IPCE curves were valued by using a solar light simulator (Newport 94063) with the air mass 1.5 global (AM 1.5G) light. Electrochemical impedance spectroscopy (EIS) was measured in range of 100 kHz to 0.1 Hz by a computer controlled potentiostat (Autolab 320, Metrohm).

## 3. Results and discussions

**Fig. 2:**
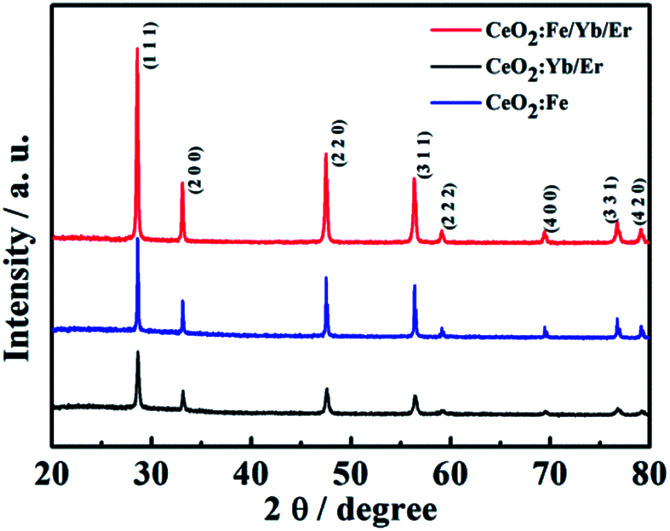
XRD patterns for the CeO_2_:Yb/Er, CeO_2_:Fe/Yb/Er and CeO_2_:Fe materials.

Fig. 2 shows the XRD patterns of different three nanomaterials. The characteristic diffraction peaks of all samples in the figure correspond to the (111), (200), (220), (311), (222), (400), (331) and (420) crystal planes of cubic phase CeO_2_ (JCPDS card no. 34-0394). And there is no other metal diffraction peaks. It can be seen that the material of CeO_2_:Fe/Yb/Er has the sharpest diffraction peak and the highest crystallinity, its 2*θ* angle that corresponding to the (111) crystal plane has shifted compared to the CeO_2_:Yb/Er material. This phenomenon demonstrates the successful doping of Fe^3+^ ions in the main system of CeO_2_ and the phase structure of CeO_2_ did not change. We can learn from the already reported literature that the doping of Fe^3+^ in the host lattice is achieved by replacing other ions or occupying the gap sites. What's more important, these two occupancies of Fe^3+^ ions in the main matrix both can change the environment around Er^3+^, thereby breaking the forbidden band transition and facilitating the f–f intra-configuration transition of rare earth ions.^18^

**Fig. 3:**
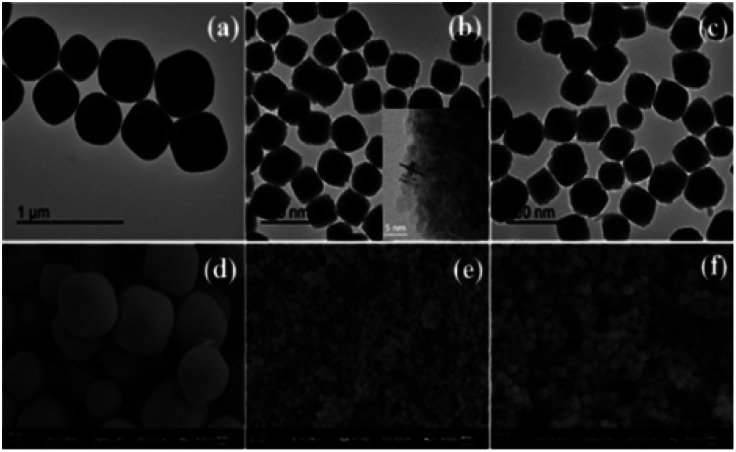
TEM images of (a) CeO_2_:Yb/Er; (b) CeO_2_:Fe/Yb/Er; (c) CeO_2_:Fe and SEM images of (d) CeO_2_:Yb/Er; (e) CeO_2_:Fe/Yb/Er; (f) CeO_2_:Fe.

SEM and TEM images characterize the morphologies of the prepared samples. As shown in Fig. 3a and d, the CeO_2_:Yb/Er sample with a particle diameter of 800 nm is composed of octahedral porous structures. Fig. 3b inset displays the HRTEM image of the sample and clearly shows that the nanoparticles are formed by agglomeration of small nanoparticles with a particle size of 3–5 nm. When the Fe^3+^ ions are introduced, the samples of CeO_2_:Fe/Yb/Er (Fig. 3b and e) and CeO_2_:Fe (Fig. 3c and f) are not only well dispersed but also uniform in size, their particle size was shrunk to about 120 nm. From the comparison of the three samples, it is observed that the doping of Er^3+^ and Yb^3+^ ions have no effect on the morphology of materials, but the Fe^3+^ doping has obviously influenced the particle size of samples.

It can be deduced that the ionic radius of Er^3+^ (0.89 Å) and Yb^3+^ (0.868 Å) have little difference with the Ce^4+^ (0.87 Å), whereas Fe^3+^ (0.64 Å) with much smaller ionic radius than Ce^4+^ (0.87 Å), which induce to hinder the growth of the crystal.^9^

**Fig. 4:**
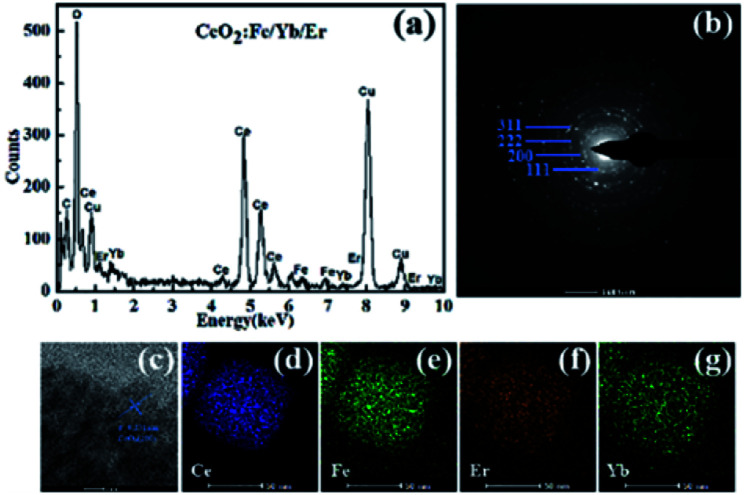
(a) EDX spectrum; (b) SAED pattern; (c) HRTEM image and (d–g) elemental mapping of the CeO_2_:Fe/Yb/Er nanomaterials.

Fig. 4 specifically shows the structural details of the CeO_2_:Fe/Yb/Er crystal. The EDX spectrum (Fig. 4a) confirms the successful doping of Er^3+^, Yb^3+^ and Fe^3+^ ions in samples.

The elemental mappings in Fig. 4d–g further prove the uniform distribution of Ce, Fe, Yb and Er in the whole nanoparticles. The SAED pattern (Fig. 4b) and HRTEM image (Fig. 4c) display the intercrystalline diffraction rings of CeO_2_:Fe/Yb/Er UCNPs and a typical 0.271 nm lattice spacing attributed to the (111) crystal plane of CeO_2_.

**Fig. 5:**
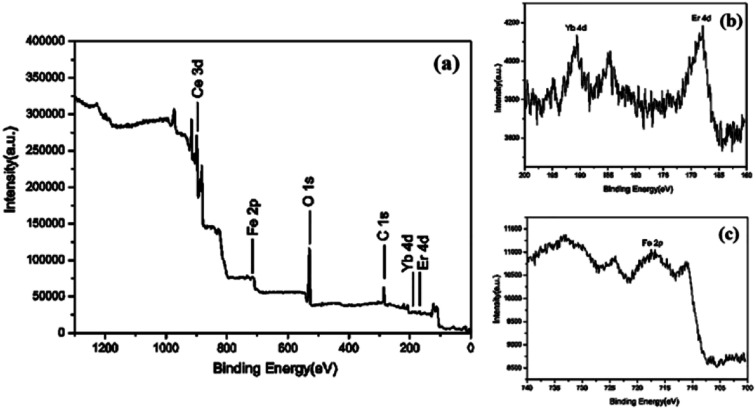
XPS spectrum of CeO_2_:Fe/Yb/Er nanomaterials (a) Yb 4d, (b) Er 4d and (c) Fe 2p.

The surface elemental composition and chemical states of CeO_2_:Fe/Yb/Er is analyzed by XPS. It can be know from the XPS survey spectrum (Fig. 5a) that the CeO_2_:Fe/Yb/Er UCNPs only contains several elements of Ce, O, Fe, Er and Yb. The core level XPS spectrum of Ce 3d is attributed to the signal binding energies of Ce^4+^ state while some of Ce^3+^ state, the binding energy peak at 902.4 eV is attributed to high concentration of Ce^3+^ and the binding energy peaks at 919.3, 900.3 eV are attributed to Ce^4+^ oxidation state.

As shown in Fig. 5b, the Yb 4d and Er 4d XPS peaks were observed at a lower binding energy position at 192.3 eV and 168.4 eV, respectively.^19–21^ XPS signals of Fe 2p (Fig. 5c) at 711 eV can be assigned to Fe 2p_3/2_ and Fe 2p_1/2_. The major peak at 530.0 eV corresponds to O_2_ and another two shoulder peaks around the main peak at 530 eV may correspond to be oxygen adsorbed water and forming hydroxyl groups. XPS proved the presence of Fe^3+^, Yb^3+^, Er^3+^, Ce^4+^ and Ce^3+^ ions in the samples.

**Fig. 6:**
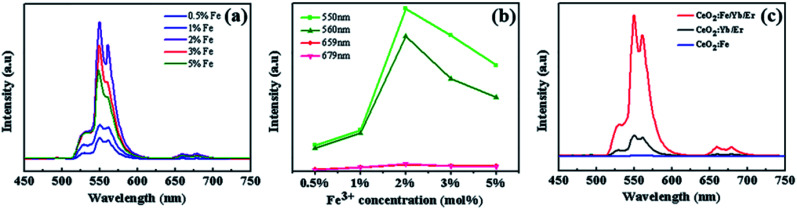
(a) The upconversion luminous spectrum of CeO_2_:Fe/Yb/Er with doped different concentrations of Fe^3+^ ion; (b) the influence of Fe^3+^ ion doping concentration on the intensity of green and red light emission; (c) the fluorescence contrast diagram of the three materials.

When the amount of Yb^3+^ and Er^3+^ is constant, Fig. 6a shows the UC luminescence spectra of CeO_2_:Fe/Yb/Er UCNPs with Fe^3+^-doping concentration from 0.5% to 5% at room temperature under the commercial continuous wave diode 980 nm laser. The upconversion emission intensities were changed with the Fe^3+^ doping. Two green emissions ranging from 517 to 532 nm and from 532 to 551 nm were deduced from the ^2^H_11/2_ → ^4^I_15/2_ and ^4^S_3/2_ → ^4^I_15/2_ transitions respectively. The red emission is observed at 659 nm and 679 nm, which due to the ^4^F_9/2_ → ^4^I_15/2_ transition of Er^3+^.^22^ It can be known that the intensity of the upconversion photoluminescence changes significantly with the increase of the Fe^3+^ ion concentration, and the luminescence intensity is the strongest at the Fe^3+^-doping concentration of 2 mol%. While the enhancement rate of green light is obviously higher than the red region (Fig. 6b). It can also be observed from the fluorescence spectra of the three materials in Fig. 6c that doping Fe^3+^ does not affect the peak position. Furthermore, the green and red emission of CeO_2_:Fe/Yb/Er (*C*_Fe_ = 2 mol%) samples are nearly 7 and 3 times stronger than those of CeO_2_:Yb/Er materials, respectively. But the CeO_2_:Fe samples without doped rare earth ions have no luminescent effect. This phenomenon is probably due to the fact that the Fe^3+^ ions with a smaller ion radius in the lattice shorten the average bond lengths of O–Ln bond, which destroys the symmetry of local crystal field around the RE ions and increases the probability of electric dipole transition, therefore the upconversion luminous intensity is obviously enhanced. But the higher concentration of Fe^3+^ may induces severe distortion of the crystal lattice, which will affects the spatial distribution of Er^3+^ ions and cause concentration quenching, which eventually induced the fluorescence radiation decreased.^12,23^ Possible reason is there may be Yb^3+^–Fe^3+^ dimer complex formation in the Yb^3+^–Er^3+^–Fe^3+^ tridoped system. The mixed electron wave functions of 3d^5^ electron-exposed Fe^3+^ ions and Yb^3+^ ions formed some new energy levels, thus triggering new energy transfer and enhancing UC emission.^18,21^

**Fig. 7:**
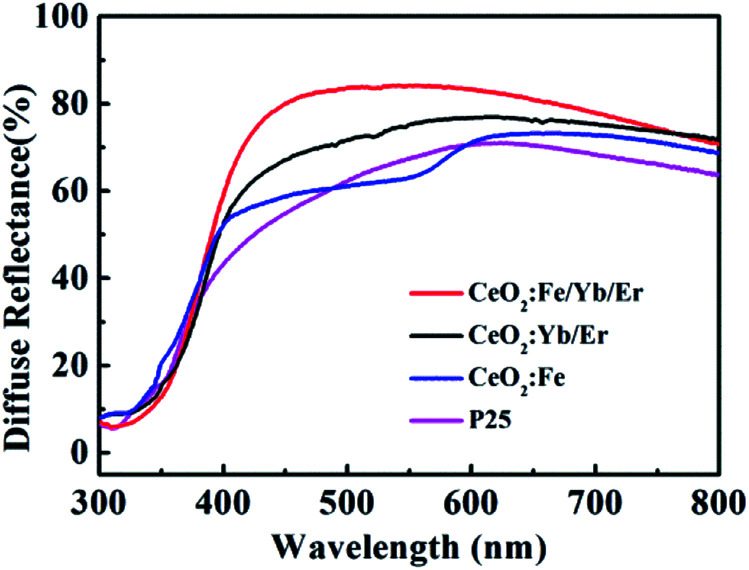
Diffuse reflectance spectra from different photoanodes.

The UV diffuses reflectance spectra of four samples (Fig. 7) display that the changes after doping with Fe^3+^ ions are clearly related to the characteristics of unsensitized photoanodes. The reflectance characteristics of the CeO_2_:Fe/Yb/Er increase distinctly compared with other materials. Furthermore, the CeO_2_:Fe/Yb/Er electrode shows the highest reflectance within the long wavelength region (550–800 nm). When sunlight is irradiated onto DSSCs, the NIR photons can be converted into visible photon by the CeO_2_:Fe/Yb/Er nanomaterial layers. It is indicated that the upconversion luminescent materials can be used as an excellent light scattering medium for expanding the optical path length in the electrode, which will improve its light capturing ability and photoelectric conversion efficiency. It is worth mentioning that the electrode coated with CeO_2_:Fe materials has no advantage in light scattering because of its no upconversion characteristics.^24^

**Fig. 8:**
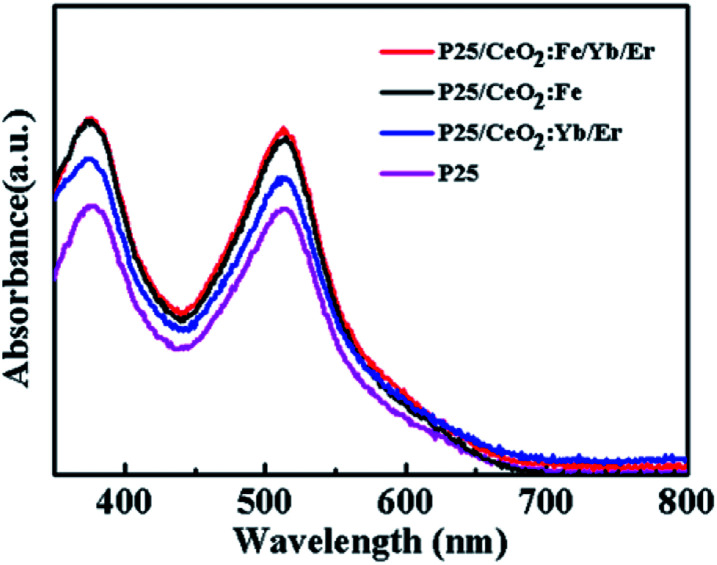
UV-Vis absorption of desorbed dye from different photoanode films.

As we all know, the dye loading capacity of the photoanode films will greatly affect the efficiency of the DSSCs. Fig. 8 is an absorption spectrum of N719 dye in a 0.1 M NaOH solution. Obviously, the photoanodes after printed materials have a significant improvement in dye adsorption compared to the pure P25 electrode. Among them, the films of CeO_2_:Fe/Yb/Er and CeO_2_:Fe have the same dye adsorption capacity substantially, and both are larger than CeO_2_:Yb/Er film. This may be attributed to the smaller particle diameter of CeO_2_:Fe/Yb/Er and CeO_2_:Fe nanomaterials than the CeO_2_:Yb/Er and the enhancement of the ability in dyes adsorbing.

**Fig. 9:**
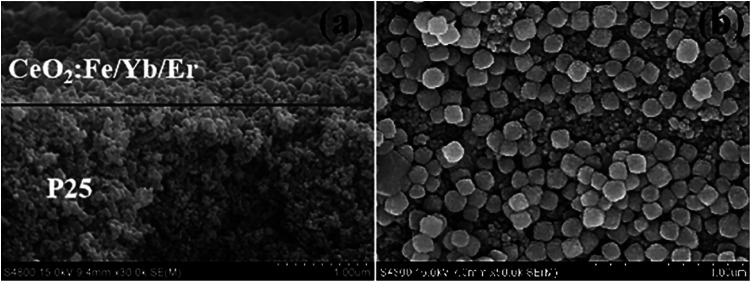
(a) SEM images of cross-section of the bilayer photoelectrode; (b) the surface for CeO_2_:Fe/Yb/Er and P25 layer.

The top and cross-section of the photoanode were characterized by SEM images. Fig. 9a shows that the photoanode of DSSCs is a typical two-layer structure, and its light scattering materials is tightly covered on the host material TiO_2_. The two layers fused to form a large assembly and the boundary is clearly visible. As can be seen in Fig. 9b, the upconversion nanomaterials are adhered to each other and are uniformly arranged on the P25 layer with the help of adhesives which is consisted of ethylcellulose and Triton X-100 terpineol solution.^25^

**Fig. 10:**
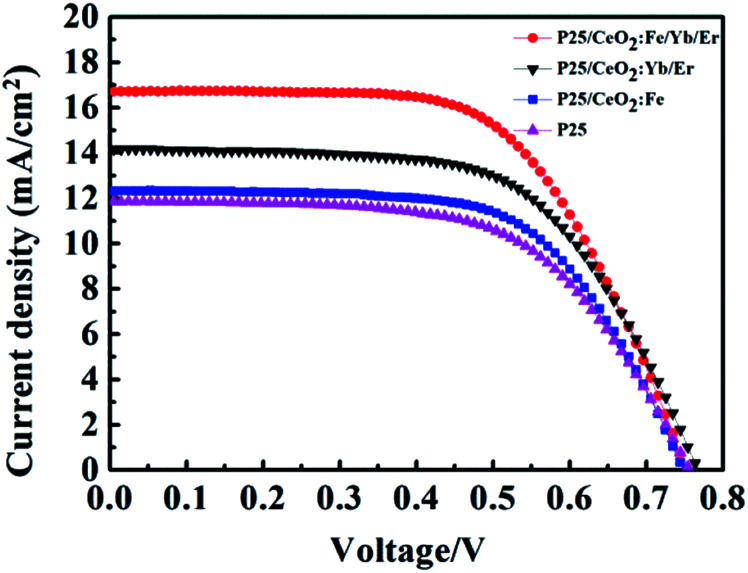
The *I*–*V* curves of DSSCs with different photoanodes.

The DSSCs coated with different materials have been tested for *I*–*V* (Fig. 10) and the photovoltaic parameters are shown in Table 1. After coating CeO_2_:Fe material on the P25 film, the short-circuit current is improved from 11.85 mA cm^−2^ which only had the TiO_2_ film up to the 12.32 mA cm^−2^ and the photoelectric conversion efficiency increased form 5.47% to 5.89%. When a layer of CeO_2_:Yb/Er up-conversion material was added to the P25 layer, *J*_es_ and *η* were further raised to 14.17 mA cm^−2^ and 6.74%, respectively. Here, the enhancement of efficiency may rely on the broadened range of absorbable spectrum, and making fuller use of near-infrared light in sunlight. What is more important, a high conversion efficiency of 7.30% and a short-circuit current of 16.70 mA cm^−2^ were achieved because the introduction of the composite material CeO_2_:Fe/Yb/Er on the TiO_2_ film as a light scattering layer. The enhancement of 33.5% than the pure P25 electrode in efficiency was directly ascribed to the dye loading capacity, light capture ability and upconversion effect of CeO_2_:Fe/Yb/Er UCNPs. Especially its stronger upconversion luminescence intensity makes the battery efficiency enhanced by 8.3% compared with the CeO_2_:Yb/Er electrode. Note that its battery efficiency is 23.9% higher than the CeO_2_:Fe battery can be further seen that the upconversion effect plays a vital role in DSSCs.

**Table 1:** Photovoltaic properties and dye loading properties of DSSCs with different photoanodes under Am 1.5 G light

	*J*_sc_ (mA cm^−2^)	*V*_oc_ (V)	FF	*η* (%)
CeO_2_:Fe/Yb/Er	16.704	0.744	0.610	7.30
CeO_2_:Yb/Er	14.167	0.763	0.613	6.74
CeO_2_:Fe	12.323	0.744	0.631	5.89
P25	11.852	0.753	0.603	5.47

**Fig. 11:**
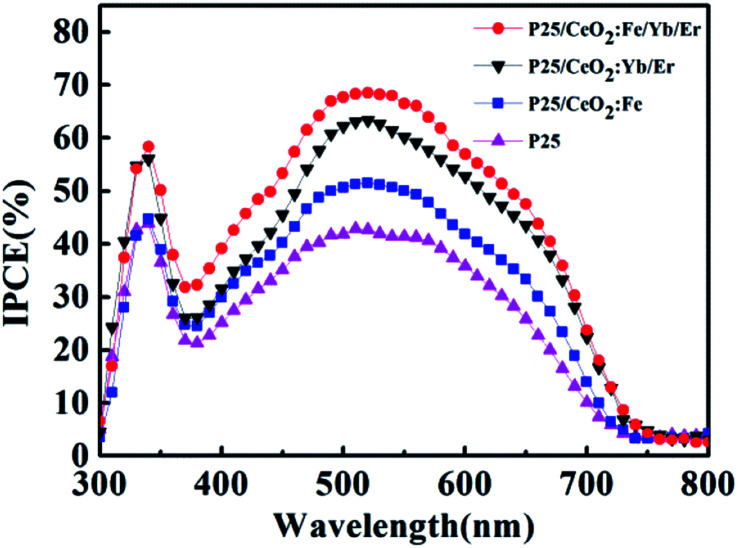
IPCE based on DSSCs for different photoanodes.

The IPCE spectrum of batteries is detailed in Fig. 11. In a certain wavelength range, the light capture ability of all photoanodes improved compared to the P25 electrode with the lowest IPCE value. It is attributed that the stronger dye absorption capacity on the particles surface will generate more photoelectrons to remarkably improve the photoelectric conversion efficiency. In addition, the UC luminescence effect is believed to enhance the light-trapping ability of the electrodes by enhancing the optical density. Herein, the CeO_2_:Fe/Yb/Er layer obtains the highest IPCE value because of its stronger up-conversion luminescence intensity. The measurement result of IPCE is consistent with *I*–*V* and UV tests.

**Fig. 12:**
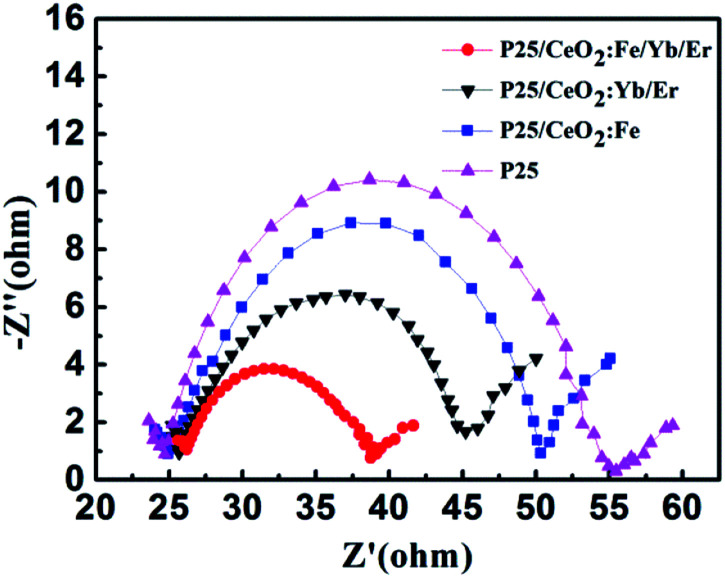
EIS Nyquist spectra for DSSCs with different photoanodes.

In order to investigate the transmission and recombination of photo-generated electrons in DSSCs, the electrochemical impedance measurements of solar cells with four different photoanodes were performed under the condition that the light intensity is 100 mW cm^−2^ and the scanning frequency ranges from 100 kHz to 0.1 Hz, the result is shown in Fig. 12. The middle large semicircle is ascribed to the recombination resistance and chemical capacitance across the photoanode/dye/electrolyte interface. The CeO_2_:Fe/Yb/Er electrode doped with Fe^3+^ ions obtains the lowest electronic recombination resistance with a value of 12.5 Ω. This incident confirmed that the increase number of dye molecules and the improvement of light utilization can reduce the impedance at the intermediate frequency, thereby increasing the amount of photo-generated electrons. Thus the purpose of heighten the photoelectric conversion efficiency of DSSCs can be achieved.

## 4. Conclusions

In conclusion, the CeO_2_:Fe/Yb/Er nanomaterials with uniform size were perfectly prepared by a hydrothermal method. To guarantee the upconversion luminescent intensity, the Fe^3+^ ions were introduced in materials to enhance the intensity by changing the symmetry of the local crystal field around the Er^3+^ ions. At the meanwhile, the behavior of incorporation Fe^3+^ ions into the host material improves the light scattering ability of this material. And it also enables materials to gain better dye loading capacity because the smaller ionic radius of Fe^3+^ shrank the lattice ruler will obtain a larger specific surface area. Additionally, the photoelectric conversion efficiency of solar cells using CeO_2_:Fe/Yb/Er UCNPs as the light scattering layer can reach 7.30%, which is 33.5% higher than the pure P25 electrodes. And its process of improving the photovoltaic performance of dye-sensitized cells is mainly accomplished by expanding the spectral absorption range and making fuller utilization of sunlight. This illustrates the great possibility and feasibility of the abundant Fe^3+^ ions on the earth applied to DSSCs. Furthermore, some explorations in this work that related to the use of transition metal ion Fe^3+^ to enhance up-conversion luminescence provide a novel idea for the improvement of upconversion materials widely used in scientific research.

## Conflicts of interest

There are no conflicts to declare.

## Supplementary Material
